# Phosphorylation of Icariin Can Alleviate the Oxidative Stress Caused by the Duck Hepatitis Virus A through Mitogen-Activated Protein Kinases Signaling Pathways

**DOI:** 10.3389/fmicb.2017.01850

**Published:** 2017-09-26

**Authors:** Wen Xiong, Wei Zhang, Wenjuan Yuan, Hongxu Du, Ke Ming, Fangke Yao, Jingying Bai, Yun Chen, Jiaguo Liu, Deyun Wang, Yuanliang Hu, Yi Wu

**Affiliations:** Institute of Traditional Chinese Veterinary Medicine, College of Veterinary Medicine, Nanjing Agricultural University, Nanjing, China

**Keywords:** icariin, phosphorylated modification, duck hepatitis virus A, oxidative stress, mitogen-activated protein kinases signaling pathways

## Abstract

The duck virus hepatitis (DVH) caused by the duck hepatitis virus A (DHAV) has produced extensive economic losses to the duck industry. The currently licensed commercial vaccine has shown some defects and does not completely prevent the DVH. Accordingly, a new alternative treatment for this disease is urgently needed. Previous studies have shown that icariin (ICA) and its phosphorylated derivative (pICA) possessed good anti-DHAV effects through direct and indirect antiviral pathways, such as antioxidative stress. But the antioxidant activity showed some differences between ICA and pICA. The aim of this study is to prove that ICA and pICA attenuate oxidative stress caused by DHAV *in vitro* and *in vivo*, and to investigate their mechanism of action to explain their differences in antioxidant activities. *In vivo*, the dynamic deaths, oxidative evaluation indexes and hepatic pathological change scores were detected. When was added the hinokitiol which showed the pro-oxidative effect as an intervention method, pICA still possessed more treatment effect than ICA. The strong correlation between mortality and oxidative stress proves that ICA and pICA alleviate oxidative stress caused by DHAV. This was also demonstrated by the addition of hydrogen peroxide (H2O2) as an intervention method *in vitro*. pICA can be more effective than ICA to improve duck embryonic hepatocytes (DEHs) viability and reduce the virulence of DHAV. The strong correlation between TCID50 and oxidative stress demonstrates that ICA and pICA can achieve anti-DHAV effects by inhibiting oxidative stress. In addition, the superoxide dismutase (SOD) and glutathione peroxidase (GSH-Px) of ICA and pICA showed significant difference. pICA could significantly inhibit the phosphorylation of p38, extra cellular signal regulated Kinase (ERK 1/2) and c-Jun N-terminal kinase (JNK), which were related to mitogen-activated protein kinases (MAPKs) signaling pathways. Ultimately, compared to ICA, pICA exhibited more antioxidant activity that could regulate oxidative stress-related indicators, and inhibited the phosphorylation of MAPKs signaling pathway.

## Introduction

Duck virus hepatitis (DVH) is an acute and lethal disease of 1–3-week old ducklings. Clinically, the symptoms are characterized primarily by rapid transmission and severe hepatitis, such as ecchymotic hemorrhage and liver necrosis (Woolcock and Crighton, 1981). As the main DVH virulence factor, duck hepatitis A virus (DHAV) which belongs to the family *Picornaviridae* and genus *Avihepatovirus* classified into three different serotypes: the traditional serotype 1 (DHAV-1; Ding and Zhang, 2007), a serotype only reported in Taiwan (DHAV-2; Tseng and Tsai, 2007), and a novel serotype isolated in eastern Asia (DHAV-3; Fu et al., 2008; Xu et al., 2012). DHAV-1 has no cross-neutralization reaction with DHAV-2 and limited cross-neutralization reaction with DHAV-3 (Kim et al., 2007; Tseng et al., 2007). In mainland China, the most virulent and widespread type is DHAV-1, with a mortality rate of up to 90% in young ducklings within 1 week of age, and thus represents a huge threat to the duck-aquaculture industry.

**FIGURE 1:**
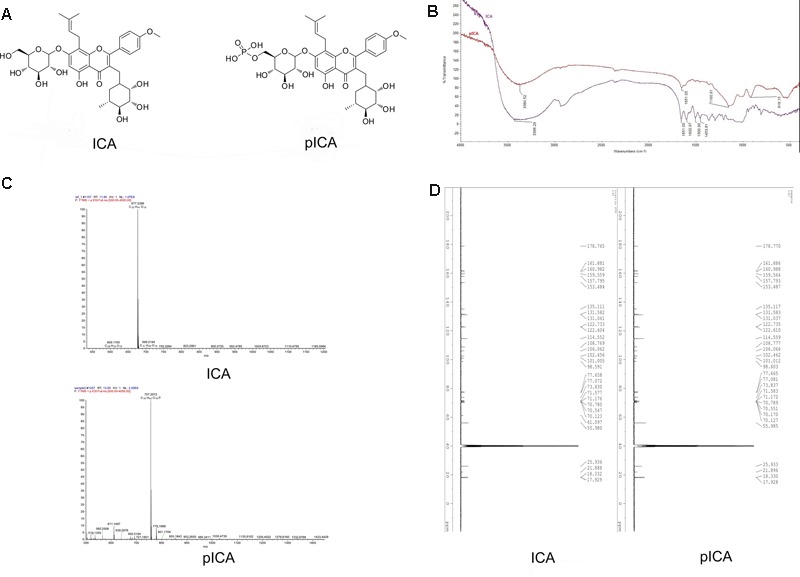
The structure of ICA and pICA. **(A)** Were the chemical Structure of ICA and pICA, **(B)** Were the FT-IR of ICA and pICA, The FT-IR spectrum exhibited characteristic absorptions for hydroxy (3384 cm^-1^), carbonyl (1652 cm^-1^), and aromatic (1610 and 1430 cm^-1^) functionalities in ICA and pICA. Furthermore, pICA also displayed extra absorption peaks at 1160.61 and 918.15 cm^-1^, corresponding to the phosphate group. **(C)** Was the HRESIMS of ICA and pICA, ICA: *m/z* 676.7400 [M + H]^+^, calcd for C_33_H_41_O_15_, 677.2396; pICA: m/z 757.2034 [M + H]^+^, calcd for C_33_H_42_O_18_P, 757.2072. **(D)** Were the ^13^C NMR of ICA and pICA. The ^13^C NMR data of pICA were similar to those of ICA, except for the C-6″′ (ICA C-6″′: δC61.10; pICA C-6″′: δC70.17), it suggested that the phosphate group was located at C-6″′.

Natural products and their derivatives have a wide range of applications in the treatment of animal viral diseases. Icariin (ICA, C_33_H_40_O_15_, **Figure 1A**), a type of flavonoid derived from plants of the genus Epimedium, exerts many biological activities and pharmacological effects, including antioxidant, antiaging, antitumor and antiosteoporosis activities (Havsteen, 2002; Kong et al., 2004; Zhang et al., 2008; Li et al., 2012). With the deepening of research, scientists are focusing not only on the biological activity of the natural product itself, but also trying to change its molecular structure to study the derived products. In the last decades, much attention has been paid to the chemical modification of natural products, which can heighten or change their biological activities (Chattopadhyay et al., 2007; Wijesekara et al., 2011; Chen et al., 2015). The phosphorylation modification, as a kind of covalent modification (Blennow et al., 2002), can change the physicochemical and biological properties of natural products such as water-solubility, antioxidative and antiviral (Yuan et al., 2005; Zhang et al., 2009; Liu et al., 2011; Deng et al., 2015). In our previous study, we successfully linked a phosphate group to icariin by the polyphosphoric acid method. Then, we performed Fourier transform infrared (FTIR, **Figure 1B**) spectroscopy analysis, high-resolution electrospray ionization mass spectrometry (HRESIMS, **Figure 1C**) analysis and nuclear magnetic resonance (NMR, **Figure 1D**) spectroscopy analysis to define its molecular structure as 8-prenylkaempferol-4′-methylether-3-rhamnosyl-7-(6″′-phosphate)-glycoside and gave it the name 6″′-phosphate icariin (pICA, C_33_H_42_O_18_P, **Figure 1A**; Xiong et al., 2015). We found that pICA is not only more water-soluble than ICA, but its anti-DHAV activity showed some difference with that of ICA. Specifically, *in vitro*, they significantly increased the cellular activity of duck embryonic hepatocytes (DEHs) after being challenged by DHAV and inhibited the replication and release of the DHAV, but pICA restrained virus replication far more effectively. Clinically, we found that ICA and pICA significantly reduced the mortality of ducklings caused by DHAV. Additional *in vivo* experiments in ducks revealed that compared to the virus control group, both ICA and pICA can reduce the amount of DHAV expression in the blood and affect various liver injury indices. As among the affected indices, the levels of serum alanine aminotransferase (ALT), alkaline phosphatase (ALP), and lactate dehydrogenase (LDH) were decreased oxidative stress indices, such as malondialdehyde (MDA) and inducible nitric oxide synthase (iNOS) were depressed, whereas the antioxidant enzymes, such as superoxide dismutase (SOD), catalase (CAT), glutathione peroxidase (GSH-Px) were increase. The Pearson’s correlation coefficients between indices of liver injury and oxidative stress showed that the indices of liver injury (ALT, ALP and LDH) were strongly correlated with the markers of oxidative stress (MDA, iNOS, GSH-Px, SOD and CAT; Xiong et al., 2014). Accordingly, we hypothesize that: ICA and pICA not only act against DVH not only though direct antiviral activity, but also though an indirect antiviral route, such as by increasing the antioxidant capacity.

In order to prove this hypothesis, we studied the effect of ICA and pICA on the oxidative stress caused by pro-oxidants to prove that ICA and pICA can indeed improve the antioxidant capacity of cells to achieve the anti-DHAV effect *in vitro* and *in vivo*. Finally, to further explore how the phosphorylation modification causes changes in drug activity, we also investigated the mechanism of different clinical effects of the ICA and pICA mediated through various signaling pathways.

## Materials and Methods

### Preparation of ICA and pICA

ICA and pICA were prepared according to our previously described method. Briefly, ICA was repeatedly purified from the extract of *Epimedium sagittatum* by column chromatography (CC) and further purified by high-pressure liquid chromatography (HPLC). The sodium trimetaphosphate - sodium tripolyphosphate method was used to prepare pICA and the crude product was purified by octadecylsilane (ODS) reversed phase column chromatography and preparative HPLC. The purity quotient of ICA and pICA were over 98%. The effect concentration of ICA and pICA were 31.25 and 156.25 μg/mL, respectively (Xiong et al., 2015).

### Reagents and Virus

Dulbecco’s modified eagle medium (DMEM; Gibco, Grand Island, NY, United States) was supplemented with penicillin 100 IU/mL, streptomycin 100 IU/mL and 10% fetal bovine serum; the maintenance medium (MM) was 1% fetal bovine serum. Dulbecco’s Hanks Balanced Salt Solution (D-Hank’s) was used for washing blood cells. The 3-(4,5-dimethylthiazol-2-yl)-2,5-diphenyltetrazolium bromide (MTT, Amresco, Solon, OH, United States) reagent was dissolved to 5 mg/mL with phosphate-buffered saline (PBS). All these reagents were filtered through a 0.22 μm syringe filters. DMEM, MM, MTT and D-Hank’s were stored at 4°C. Heparin sodium was dissolved with physiological saline to a concentration of 2 mg/mL. Other chemicals used in the experiments were analytical grade.

Hinokitiol (HK, Tokyo Chemical Industry, Tokyo, Japan), also known as 4-isopropyltropolone, was used as the pro-oxidant. Normal saline containing 0.3% Tween-80, 0.5% glycerinum and 0.1% dimethyl sulfoxide was used to dissolve HK and also to treat the ducklings. Hydrogen peroxide solution (Lot no.H1009) was bought from Sigma–Aldrich (Saint Louis, MO, United States).

The DHAV (strain *LQ_2_*) used in the challenge experiments was supplied by the Shandong Institute of Poultry (Shandong, China) and was stored at -70°C.

### *In Vivo* Experiment

#### Ethics Statement

All animal experiments conformed to the Guide for the Care and Use of Laboratory Animals published by the US National Institutes of Health (NIH Publication, Eighth edition, 2011) and were approved by the Nanjing Agricultural University Animal Care Committee. To ameliorate suffering, animals that were not expected to survive were humanely euthanized. All procedures were performed in compliance with the AVMA Guidelines for the Euthanasia of Animals (2013 Edition).

#### Animal Grouping and Treatment

A total of 480 two-day-old Cherry Valley ducklings (purchased from Nanjing Tangquan Poultry Farm) were randomly divided into eight groups: the normal groups (BC, VC, ICA and pICA) and the intervention groups (HK, VC-i, ICA-i and pICA-i). The treatment for each group of birds was as follows: from day 1 to day 3, ducklings in the BC, VC, ICA and pICA groups were injected with 0.75% normal saline (HK excluded, the solvent is consistent with that of HK group, containing 0.3% Tween-80, 0.5% glycerinum and 0.1% dimethyl sulfoxide); ducklings in the HK, VC-i, ICA-i and pICA-i groups were injected with HK (80 mg/kg of duckling). At the 4th day, ducklings in the BC and HK groups were injected 0.75% normal saline, other groups were injected DHAV; from day 4 to day 11, ducklings in the ICA, ICA-i, pICA and pICA-i groups were given water with ICA or pICA (31.25 mg/Kg of duckling, *SID*), separately, the others were given water alone.

#### Dynamic Detection

After the challenge with DHAV, five blood samples from each group were taken at the 4th, the 8th, and the 54th hour. The ducklings status were monitored at the 12th, 24th, 30th, 36th, 48th, 60th, 72nd, 96th, 120th, 144th, 168th and 192nd hour. The mortality rate of each group was calculated until no dead duckling was found. However, the number of the ducklings from which blood samples were taken (15 feathers per group) was not included in the calculation of the mortality rate. Surviving ducklings after assessing rehabilitation were sent to the Nanjing Tangquan Poultry Farm to be bred in accordance with National regulation. Disposal of the dead ducklings was executed in accordance with the standard biosafety procedure instituted by the local authorities.

#### Evaluation of Oxidative Indices *In Vivo*

The indices for the evaluation of oxidative stress, including SOD, MDA, CAT and GSH-Px in the serum of the ducklings at the 8th and the 54th hour were determined using commercially available kits (Nanjing Jiancheng Bioengineering Institute, China).

### *In Vitro* Experiment

#### Preparation of Duck Embryonic Hepatocytes (DEHs)

Livers were removed from 15-day-old duck embryos under sterile conditions, followed by gallbladder evisceration. The livers were washed three times with D-Hank’s. The tissue was minced with eye scissors and washed three times with D-Hank’s. Liver tissue was digested with a solution of 0.2% trypsin at 37°C for 4–6 min. As soon as the trypsin was completely absorbed, the tissue was rinsed three times with D-Hank’s and an appropriate amount of DMEM growth medium was added. The seeding cell density was adjusted to about 1.0 × 10^6^ cell/mL. Cells were incubated at 37°C in a humid atmosphere with 5% CO_2_, and the growth medium was replaced after 24 h. The DMEM was removed when the duck embryo hepatocytes grew into a monolayer.

### Hydrogen Peroxide (H_2_O_2_) Induced Oxidative Stress in DEHs

A 100 μL volume of H_2_O_2_ (serially diluted by twofold in MM from 1 mol/L) was added to each well of the 96-well culture plate containing the DEHs monolayer, thus obtaining 6 concentrations for each. At the same time, the cell control (CC) group was set. The plate was cultured for 2 or 4 h. Then, the H_2_O_2_ solution was removed and the monolayer was washed three times with D-Hank’s. Subsequently, 100 μL of MM was added to each well and the cells were cultured for another 48 h, the MTT method was used to determine the DEHs viability.

#### Anti-DHAV Activity of ICA and pICA after H_2_O_2_ Treatment

As in the *in vivo* experiments, the treatments are divided into two groups: the normal groups and the intervention groups. In the normal groups (CC, VC, ICA and pICA), 100 μL of DHAV was added into the wells containing the monolayer of DEHs, while in the cell control (CC, not contain DHAV) and virus control (VC, contain DHAV) wells were reserved. Then, the plate was incubated for 2 h, and subsequently the virus solution was removed and the plate was washed three times with D-Hank’s. A 100 μL dilution of ICA and pICA was separately added into the test wells (6 wells in parallel). The plate was further incubated for 48 h. In the intervention groups (H_2_O_2_, VC-i, ICA-i, pICA-I group), 100 μL of H_2_O_2_ was added into the wells containing the monolayer DEHS. After 2 h incubation, the H_2_O_2_ was discarded and the wells were washed three times with D-Hank’s, 100 μL DHAV was added into the wells containing the monolayer of DEHS, while the H_2_O_2_ treatment control (H_2_O_2_, not contain DHAV) wells were reserved. Then, the plate was incubated for 2 h and subsequently the virus solution was removed and the plate was washed three times with D-Hank’s, the virus control was reserved for the intervention treatment (VC-i). A 100 μL dilution of ICA (ICA-i) and pICA (pICA-i) was then separately added into the test wells (6 wells in parallel). The plate was further incubated for 48 h. The MTT assay was used to determine the cell viability of each DEHs group.

#### The Impact on the Virulence of the DHAV

In the normal groups (CC, VC, ICA and pICA), 400 μL of DHAV was added into the wells of a 24-well culture plate containing the monolayer DEHs, while the cell control (CC, not contain DHAV) and virus control (VC, contain DHAV) wells were reserved. After being incubated for 2 h, the wells containing the DEHs monolayer were washed three times with D-Hank’s. Then, both ICA and pICA, at the most effective concentrations, were added into wells of the ICA and pICA groups; MM was added into the wells of the CC and VC groups, three wells each group and 400 μL per well. Subsequently, the plate was incubated for 30 h. Then, the 24-well plate was washed three times with D-Hank’s to remove the remaining drugs and 400 μL of MM was added into each well. Afterward, the plate was further incubated for 24 h. In the intervention groups (H_2_O_2_, VC-i, ICA-i, pICA-i group), 400 μL H_2_O_2_ was added into the wells of the 24-well culture plate containing the monolayer DEHs. Following a 2 h incubation period, and the wells were washed three times with D-Hank’s, the rest of the process was the same as the treated normal groups. The corresponding groups were renamed as H_2_O_2_, VC-i, ICA-i and pICA-i. Also, the DHAV from each well was collected sterilely. The 50% tissue culture Infective Dose (TCID_50_) of the DHAV of each group was measured using the Reed–Muench assay.

#### Evaluation of the Oxidative Stress Indices *In Vitro*

A 1.6 mL volume of DHAV was added into the 6-well culture plate containing the DEHs monolayer, while the cell control (CC, not contain DHAV) and virus control (VC, contain DHAV) wells were reserved. The plate was incubated for 2 h, then the virus solution was removed, and the plate was washed three times with D-Hank’s. Subsequently, 1.6 mL of the ICA and pICA dilutions were separately added into the test well (three wells in parallel), as the most effective antiviral concentration. The 6-well culture plate was incubated for 48 h and following this incubation, all samples were trypsinized and collected for testing the levels of SOD, MDA, CAT, GSH and GSH-PX.

#### Western Blot Analysis of the Relative Protein Expression

Duck embryonic hepatocytes were harvested and suspended into 80 μL of lysis buffer containing protease inhibitors (Beyotime, China) and the concentration of the protein extract was determined using the BCA protein assay kit (Beyotime). Sixty micrograms of protein were diluted in sample loading buffer and heated at 95°C for 5 min. The denatured proteins were separated by 12% sodium dodecyl sulfate–polyacrylamide gel electrophoresis and transferred to polyvinylidene difluoride membranes. The membranes were blocked for 40 min at room temperature in tris-buffered saline containing 5% BSA and 0.1% tween 20, followed by overnight incubation at 4°C, separately, with specific primary antibodies (anti SOD, anti GSH-px, anti JNK, anti p-JNK, anti ERK1/2, anti p-ERK1/2, anti-p38, anti p-p38, or anti β-actin), which detect proteins involved in various cell signaling pathways. The membranes were washed and incubated with the appropriate secondary antibody (Thermo Pierce, Rockford, IL, United States) at room temperature for 1 h. Blots were visualized using a standard enhanced chemiluminescence system (Bio-Rad Labs., Hercules, CA, United States).

### Statistical Analysis

Correlations among the markers of oxidative stress and liver injury were determined using Pearson’s correlation coefficient. Comparisons among the experimental groups were made using one-way analysis of variance (ANOVA) and differences between specific groups were determined using the Duncan’s Multiple Range Test. Results are expressed as the mean ± standard deviation for five ducklings in each group. The difference in the survival rate was determined using the χ2 test. Differences were considered statistically significant at *p* < 0.05. All the statistical analyses were conducted using the SPSS Software Package version 20.0 (IBM, Armonk, NY, United States).

## Results

### Comparison of Clinical Effects between ICA and pICA

The curative effect of ICA and pICA are presented in **Table 1**. The distribution of the groups according to the observed mortality from low to high is as follows: BC = HK < pICA < pICA-i = ICA-i < ICA < VC < VC-i. Compared with the VC group, the ICA group (64.4%) and pICA group (51.1%) were significantly lower than that of the VC group (80.0%). Additionally, compared with the VC-i group, the pICA-i group (62.2%) and ICA-i group (62.2%) were significantly lower than that of the VC-i group (91.1%). Also, among the drug treatment groups, the pICA group was significantly lower than ICA group.

**Table 1:** The clinical curative effect of ICA and pICA in intervention experiment.

Group	Samples (feathers)	Final deaths (feathers)	Mortality rate (%)
BC	45	0	0.0^a^
HK	45	0	0.0^a^
VC	45	36	80.0^d^
VC-i	45	41	91.1^d^
ICA	45	29	64.4^c^
ICA-i	45	28	62.2^c^
pICA	45	23	51.1^b^
pICA-i	45	28	62.2^c^

*Data within a column without the same superscripts (a–d) differ significantly (*p* < 0.05).*

**FIGURE 2:**
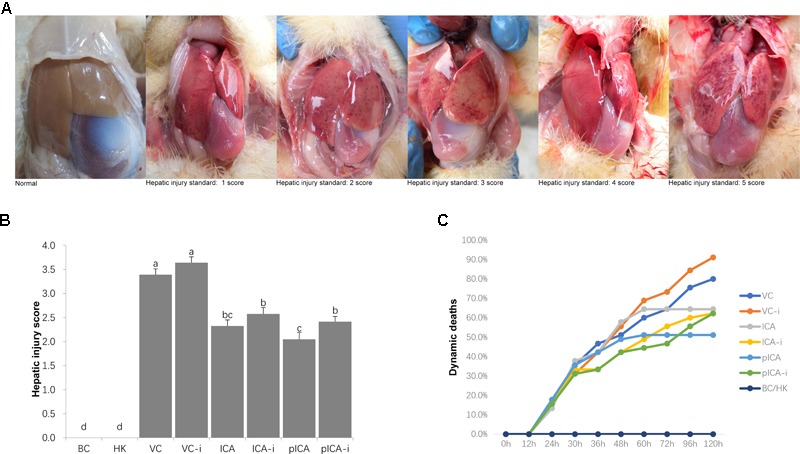
The hepatic injury score and the dynamic deaths of each group. **(A)** Was the standards score of corresponding hepatic injury, hepatic injury standards with 0–5 score to represent severity from low to high. The scores were determined according to the feature below: 0 point, healthy ducklings (BC group). 1 point, a slight hemorrhagic spot or extravasated blood. 2 points, a few hemorrhagic spot (less than 20 spots). 3 points, a mass of hemorrhagic spot (more than 20 spots meanwhile less than 100 spots). 4 points, diffuse hemorrhagic spot (almost the whole hepar). 5 points, diffuse hemorrhagic clot (the whole hepar). **(B)** Was the average hepatic injury score of each group, the order of the average score of each group from high-to-low was: VC-i > VC > ICA-i > pICA-i > ICA > pICA, (groups BC and HK are not included), ^a-d^ Bars in the figure without the same superscripts differ significantly (*p* < 0.05). **(C)** Was the dynamic deaths of each group. The death peak in each group of ducklings occurred between the 24th and the 32th hour. The ducks in the ICA and pICA groups stopped dying after 72 h of treatment, while the ducklings in the groups treated with ICA-i and pICA-i continued to die after 120 h of treatment.

The hepatic injury scores of the ducklings in each group are presented in **Figure 2**. The livers shown in **Figure 2A** were used as a hepatic injury standards with 1–5 score, respectively. In particular, there was no pathological change occurred in the liver of ducklings in the BC group. The order of the average score of each group from high-to-low was: VC-i > VC > ICA-i > pICA-i > ICA > pICA, (groups BC and HK are not included). All the drug groups were significantly lower than the VC and VC-i groups. Specifically, the ICA and pICA groups were significantly lower than the corresponding ICA-i and pICA-i groups (**Figure 2B**). Also, the results shown in **Figure 2C** revealed the dynamic deaths in each group. The death peak in each group of ducklings occurred between the 24th and the 32th hour. It is noteworthy that the ducks in the ICA and pICA groups stopped dying after 72 h of treatment, while the ducklings in the groups treated with ICA-i and pICA-i continued to die after 120 h of treatment.

### Comparison of the Antioxidant Effects of ICA and pICA *In Vivo*

**FIGURE 3:**
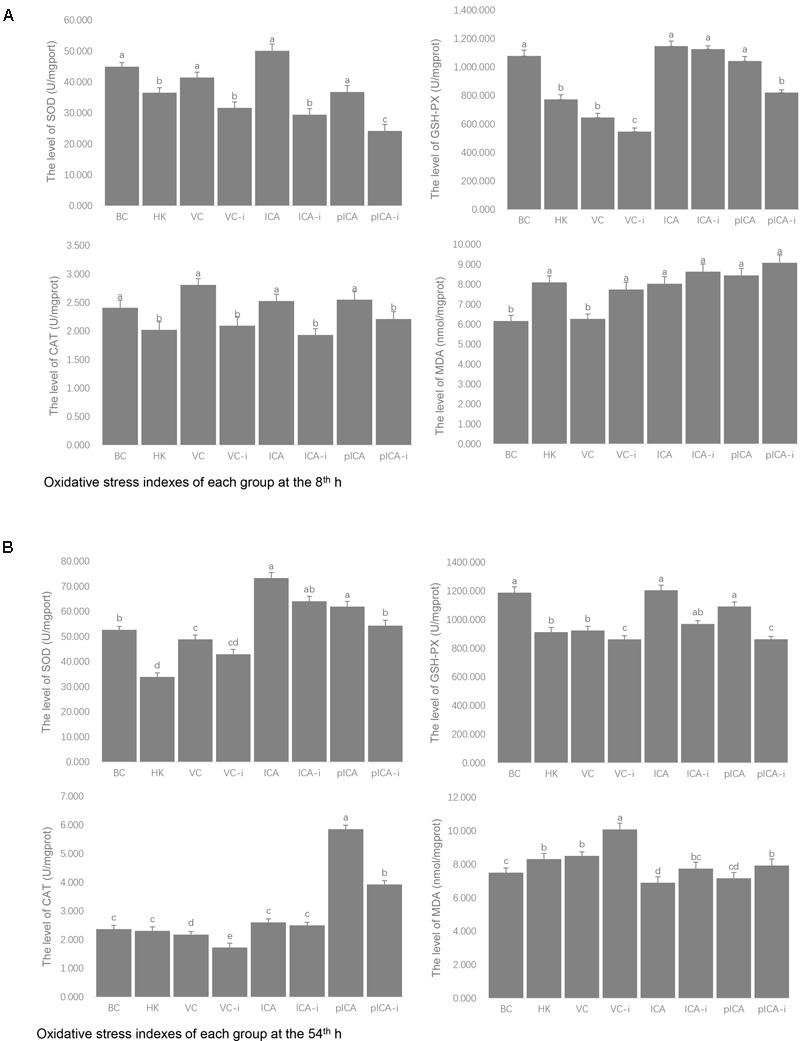
The evaluation of the levels of oxidative stress markers in each group. **(A)** Was the evaluation of the levels of oxidative stress markers at 8th hour, the SOD, GSH-Px and CAT levels in the normal group were higher than those detected in the intervention group, whereas the MDA levels were lower. **(B)** Was the evaluation of the levels of oxidative stress markers at 54th hour, the effect of pICA was significantly stronger than that of pICA-i and there was no significant difference between the effect of ICA and ICA-i in the levels of CAT, SOD and GSH. ^a-e^ Bars in the figure without the same superscripts differ significantly (*p* < 0.05).

The evaluation of the levels of oxidative stress indexes in each group is shown in **Figure 3**. At the 8th h, the SOD, GSH-Px and CAT levels in the normal group were higher than those detected in the intervention group, whereas the MDA levels were lower (**Figure 3A**). A similar trend was observed on the 54th h. In particular, the effect of pICA was significantly stronger than that of pICA-i and there was no significant difference between the effect of ICA and ICA-i in the levels of CAT, SOD and GSH (**Figure 3B**).

### Pearson’s Correlation Coefficients between Mortality Rate and Oxidative Stress Index

The correlation coefficients among the measured indices of mortality and oxidative stress index are listed in **Table 2**. These results reveal that the mortality rate was negatively correlated with the levels of GSH-Px, SOD and CAT, whereas it was positively correlated with the MDA level. In particular, the mortality rate was significantly associated with the levels of GSH-Px and MDA (*p* < 0.05). We also found that there was extremely high positive relevance between GSH-Px and SOD (*p* < 0.01).

**Table 2:** Pearson’s correlation coefficients between mortality rate and oxidative stress index.

	Mortality rate	GSH-PX	SOD	MDA	CAT
Mortality rate	1	-0.544	-0.743	0.896^∗^	-0.809
GSH-PX		1	0.867^∗^	-0.772	0.305
SOD			1	-0.905^∗^	0.306
MDA				1	-0.574
CAT					1

*^∗^*p* < 0.05. 0.8–1.0 extremely correlation, 0.6–0.8 strong correlation, 0.4–0.6 moderate correlation, 0.2–0.4 weak correlation, 0.2–0.0 very weakly correlation or no correlation.*

### Effects of H_2_O_2_ on Oxidative Stress Damage to DEHs

The evaluation of the effective concentration of H_2_O_2_ causing oxidative damage to DEHs is shown in **Table 3**. After 2 h and 4 h treatment with H_2_O_2_, the cell viability was 50% when the concentration of H_2_O_2_ was 1.953 μmol/mL, and reached 85% at the concentration of 0.977 μmol/mL. In this interval, cell survival was linearly dependent on the concentration of H_2_O_2_. This indicates that H_2_O_2_ causes oxidative stress damage to DEHs within a certain concentration interval. After the experiment, we selected a concentration of 1.5 μmol/mL to achieve a cell viability of 50–85% in the followed experiments.

**Table 3:** H_2_O_2_ effective concentration in DEHs.

Concentration (μmol/mL)	*A*_570_		Cell viability (% of control)	
	2 h	4 h	2 h	4 h
125.000	0.074 ± 0.003^e^	0.120 ± 0.002^f^	11.4	15.3
62.500	0.076 ± 0.003^e^	0.126 ± 0.002^f^	11.7	16.0
31.250	0.069 ± 0.002^e^	0.130 ± 0.003^f^	10.7	16.7
15.625	0.074 ± 0.004^e^	0.119 ± 0.003^f^	11.3	15.2
7.813	0.079 ± 0.003^e^	0.152 ± 0.003^f^	12.2	19.5
3.906	0.137 ± 0.003^d^	0.216 ± 0.006^d^	21.1	27.6
1.953	0.332 ± 0.002^c^	0.462 ± 0.013^c^	51.4	59.1
0.977	0.572 ± 0.004^b^	0.669 ± 0.011^b^	88.4	85.8
0.488	0.654 ± 0.011^a^	0.789 ± 0.007^a^	101.1	101.1
CC	0.646 ± 0.003^a^	0.031 ± 0.012^a^		

*Data within a column without the same superscripts (a–f) differ significantly (*p* < 0.05).*

### Anti-DHAV Activity of ICA and pICA after H_2_O_2_ Treatment

**FIGURE 4:**
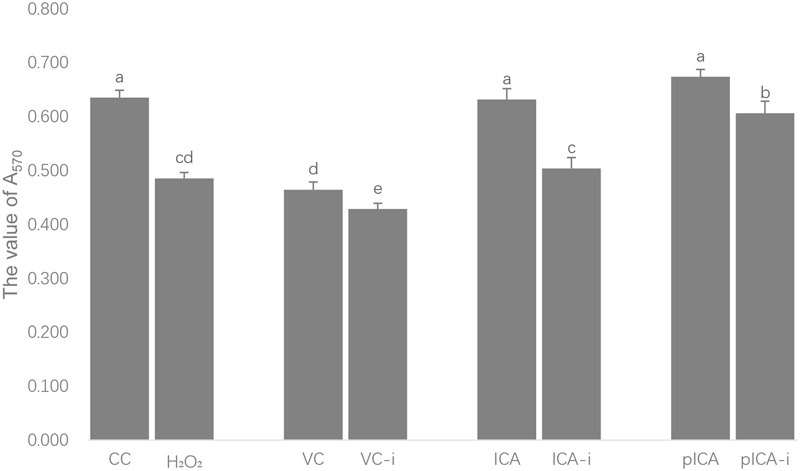
Anti-DHAV activity of ICA and pICA after H_2_O_2_ treatment. Among the normal groups, the CC, ICA and pICA groups exhibited significantly higher antiviral activity than that in the VC group. Among the intervention groups, the H_2_O_2_, ICA-i and pICA-i groups showed significantly higher antiviral activity than that of the VC-i group, while the pICA-i group showed significantly higher activity than those of the H_2_O_2_ and ICA-i groups. On the other hand, the CC, VC, ICA and pICA groups exhibited significantly lower activity than those of H_2_O_2_, VC-i, ICA-i and pICA-i groups. ^a-e^ Bars in the figure without the same superscripts differ significantly (*p* < 0.05).

The measurements of the antiviral activities of ICA and pICA after H_2_O_2_ treatment are shown in **Figure 4**. Among the normal groups, the CC, ICA and pICA groups exhibited significantly higher antiviral activity than that in the VC group. Among the intervention groups, the H_2_O_2_, ICA-i and pICA-i groups showed significantly higher antiviral activity than that of the VC-i group, while the pICA-i group showed significantly higher activity than those of the H_2_O_2_ and ICA-i groups. On the other hand, the CC, VC, ICA and pICA groups exhibited significantly lower activity than those of H_2_O_2_,VC-i, ICA-i and pICA-i groups.

### The Virulence of the DHAV after Intervention Treatment

The TCID_50_ of the DHAV in the VC, ICA, pICA, VC-i, ICA-i and pICA-i groups was 10^-3.626^, 10^-2.543^, 10^-2.289^, 10^-5.294^, 10^-3.883^, and 10^-3.386^, respectively.

### Comparison of the Antioxidant Effects of ICA and pICA *In Vitro*

**FIGURE 5:**
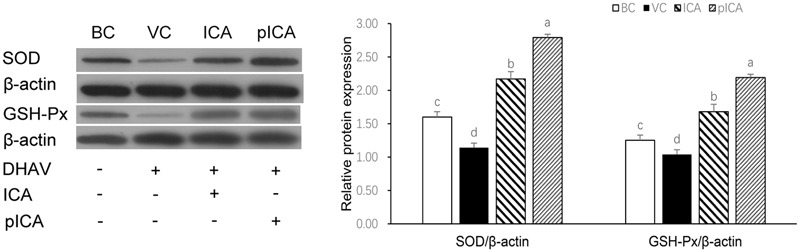
The changes of SOD and GSH-Px protein expression *in vitro.* The relative expression of the SOD and GSH-PX proteins in the BC group was significantly higher than that in the VC group. the expression in the ICA and pICA group was significantly higher than that in the BC group. The expression in the pICA group was also significantly higher than that in the ICA group. The effect concentration of ICA and pICA were 31.25 μg/mL and 156.25 μg/mL, respectively. ^a-d^ Bars in the figure without the same superscripts differ significantly (*p* < 0.05).

The levels of the oxidative stress-related indicators in cells treated with either ICA or pICA *in vitro* are shown in **Table 4**. The levels of MDA and NOS in the BC group were significantly lower than those in the VC group, whereas the levels of SOD, CAT, GSH and GSH-PX were significantly higher than those in the VC group. In the drug treatment groups, the level of MDA in the groups treated with ICA or pICA was significantly lower than that in the VC group, but was significantly higher than that in the BC group. However, although no significant difference was found in the NOS level, the levels of SOD, CAT, GSH and GSH-PX were significantly higher than those in the VC group. In addition, there was also a significant difference in the groups treated with pICA. For instance, the levels of SOD and GSH-PX were significantly higher in the group treated with pICA than those in the ICA treated group. The relative expression of the SOD and GSH-PX proteins are presented in **Figure 5**, which shows that their expression in the BC group was significantly higher than that in the VC group. Additionally, the expression in the ICA and pICA groups was significantly higher than that in the BC group. Although, the expression in the pICA group was also significantly higher than that in the ICA group.

**Table 4:** Evaluation indices of oxidative *in vitro*.

Index	BC	VC	ICA	pICA
SOD (U/mgport)	50.025 ± 0.376^c^	36.316 ± 0.738^d^	53.204 ± 0.269^b^	55.339 ± 0.570^a^
MDA (nmol/mgprot)	9.668 ± 0.731^b^	12.053 ± 0.950^a^	4.198 ± 0.658^c^	3.833 ± 0.316^c^
NOS (U/mgprot)	22.145 ± 0.192^b^	25.923 ± 0.712^a^	23.922 ± 0.747^ab^	23.695 ± 0.840^ab^
CAT (U/mgprot)	20.224 ± 0.336^a^	18.555 ± 0.224^b^	20.667 ± 0.495^a^	19.998 ± 0.382^a^
GSH (μmol/L)	8.885 ± 0.884^a^	2.435 ± 0.217^c^	5.006 + 0.513^b^	6.636 ± 0.801^b^
GSH-PX (U/mgprot)	110.903 ± 2.205^c^	75.298 ± 1.275^d^	125.013 ± 1.267^b^	142.872 ± 3.378^a^

*Data within a column without the same superscripts (a–d) differ significantly (*p* < 0.05).*

### Pearson’s Correlation Coefficients between Virulence and Oxidative Stress Index

The calculated correlation coefficients among the indices of virulence and oxidative stress index are listed in **Table 5**. The results shown in this table indicate that the virulence was negatively correlated with the levels of GSH-Px, SOD, CAT and GSH, whereas it was positively correlated with the MDA level. In particular, regarding the negative correlation, the GSH-Px and SOD showed strong correlation with the virulence, CAT showed extremely high correlation and the GSH showed moderately high correlation. Regarding the positive correlation, the virulence showed extremely high correlation with the MDA level. In addition, the SOD showed significant correlation with the MDA level (*p* < 0.05).

**Table 5:** Pearson’s correlation coefficients between TCID_50_ and oxidative stress.

	TCID_50_	GSH-PX	SOD	GSH	MDA	CAT
TCID_50_	1	-0.674	-0.780	-0.567	0.818	-0.967
GSH-PX		1	0.988	0.991	-0.976	0.840
SOD			1	0.958	-0.998^∗^	0.914
GSH				1	-0.937	0.758
MDA					1	-0.938
CAT						1

*^∗^*p* < 0.05. 0.8–1.0 extremely correlation, 0.6–0.8 strong correlation, 0.4–0.6 moderate correlation, 0.2–0.4 weak correlation, 0.2–0.0 very weakly correlation or no correlation.*

### Western Blot Analysis of Components of the MAPKs Signaling Pathways

**FIGURE 6:**
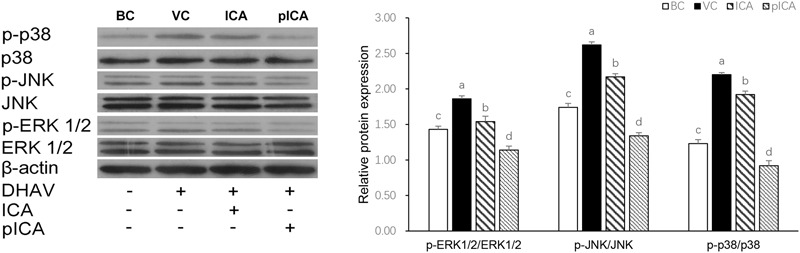
The changes of MAPKs signaling pathways relative protein expression *in vitro.* The expression of p-ERK 1/2, p-JNK and p-p38 proteins in the VC group was significantly higher than those that detected in other groups. In the drug-treatment group, the expression of these proteins in the group treated with pICA was significantly lower than their expression in the ICA treated group. The effect concentration of ICA and pICA were 31.25 and 156.25 μg/mL, respectively. ^a-d^ Bars in the figure without the same superscripts differ significantly (*p* < 0.05).

As shown in **Figure 6**, the expression of p-ERK 1/2, p-JNK and p-p38 proteins in the VC group was significantly higher than those that detected in other groups. In the drug-treatment group, the expression of these proteins in the group treated with pICA was significantly lower than their expression in the ICA treated group.

## Discussion

Numerous serious outbreaks of DVH in ducklings have been reported, which have caused serious economic losses to the commercial duck industry (Kamomae et al., 2017). Currently, modified live virus vaccines, which are attenuated by serial passages in chicken embryos, are available for controlling DHAV-1 infection in ducks (Woolcock and Crighton, 1979). However, there are a variety of inconveniences in the actual application of the vaccine. For instance, the vaccine requires full cold chain preservation. In China, which is dominated by small-scale farms run by individuals, it is difficult for them to have the right conditions for the preservation of vaccines and their appropriate use. Moreover, the vaccines can only be injected one by one, which poses a great challenge to breeders in practice. Accordingly, developing an anti-DHAV drug that is easy to store and use is the most economical strategy for dealing with these crises in China and other countries with similar situation.

In our previous studies on the anti-DVH activity of natural products and their derivatives, we found that chemical and non-chemical modifications of natural products showed some differences. For example, the anti-DHAV activity and the hepatic injury alleviating effect of the sulfated Bush Sophora Root polysaccharide were better than those of Bush Sophora Root polysaccharide, although both have the same ability to scavenge free radicals (Chen et al., 2014a,b). In the previous study, we found that several indices of liver injury caused by DHAV (ALT and LDH) were significantly and negatively correlated with certain indices of antioxidant capacity (GSH-Px, CAT, and SOD), and that SOD might be one of the most important factors for limiting the degree of liver injury (Xiong et al., 2014). ICA and pICA have antioxidant activities and are hepatoprotective, as indicated by the reduction of the levels of several serum liver injury markers and the increase of the levels of antioxidative enzymes. Accordingly, we surmise that ICA and pICA might be able to protect against the DHAV by raising the duck’s antioxidant capacity.

In order to verify this hypothesis, we designed some intervention experiments where hinokitiol or H_2_O_2_ were used as pro-oxidative agents *in vivo* and *in vitro* (Prieto et al., 2014). The purpose of these experiments is to counteract the antioxidant activity of ICA and pICA to determine whether the ICA and pICA really have an antioxidant effect. Failure to detect any significant difference between the intervention group and the normal group would indicate that the anti-DHAV pathway of the ICA or pICA have nothing to do with its antioxidant capacity. On the other hand, detection of significant difference between them would prove that the ICA or pICA indeed exert their anti-DHAV effect through antioxidant pathway(s). The *in vivo* experiments reveal some interesting results. First, the mortality rates were widely divergent: the mortality rate of the VC-i group (91.1%) was significantly higher than that of the VC group (80.0%), and that of the pICA-i group (62.2%) was significantly higher than that of the pICA group (51.1%). However, the mortality rate of the ICA group (62.2%) was comparable to that of ICA group (64.4%). These results suggest that the intervention agents can significantly increase the mortality of the VC group and the pICA group, whereas no significant effect on the ICA group was detected. Second, by measuring the dynamic mortality rate, we found that the ICA and pICA groups stopped dying at 60 h after the challenge with DHAV, whereas the hinokitiol treated groups (ICA-i and pICA-i) continued to die until 120 h. In addition, we established the hepatic injury score to measure the degree of clinical liver injury. Consistent with the mortality rate findings, the average hepatic injury score of the pICA-i group (2.41) was significantly higher than that of the pICA group (2.04), but that of the ICA-i group (2.57) was not significantly different from that of the ICA group (2.32). Third, the evaluation of plasma oxidative stress indices revealed some noteworthy results. For instance, whether at the 8th hour or the 54th hour, the levels of GSH-Px, SOD and CAT in the intervention groups (HK, VC-i, ICA-i and pICA-i) were lower than those of the corresponding normal groups (BC, VC, ICA and pICA), whereas the MDA level was higher in the intervention groups compared with the normal groups. In particular, the pICA group was significantly higher than the pICA-i group, but there was no significant statistical difference between the ICA group and the ICA-i group in the levels of CAT, SOD, and GSH-Px at the 54th hour.

The change trend of antioxidant enzymes was consistent with the determined mortality rate. The pICA group was significantly lower than the pICA-i group and there was no significant difference between the ICA group and the ICA-i group in the levels of CAT, SOD, and GSH-Px. Accordingly, we surmise that there is a relationship between oxidative stress index and mortality. Subsequent Pearson’s correlation analysis between the groups’ results proved our hypothesis (**Table 2**). We found that there was a strong correlation between mortality with antioxidant enzymes, especially between mortality and MDA (*p* < 0.05). These results demonstrated that oxidative stress was directly related to mortality and was one of the important pathways involved in DHAV pathogenesis. However, especially under intervention conditions, ICA and pICA showed different antioxidant activity and the corresponding difference in mortality. Therefore, it is necessary to further explore the reasons for such difference between them *in vitro*.

The direct anti-DHAV effect of the drugs on DEHs undergoing H_2_O_2_-induced oxidative stress, *in vitro*, further confirmed our hypothesis. First, the cytotoxic effects of various concentrations of H_2_O_2_ on DEHs, shown in **Table 3**, revealed that regardless of whether the DEHs were incubated with H_2_O_2_ for 2 or 4 h, the cell viability was more than 50% at 1.953 μmol/mL, and the cell viability increased linearly or even higher than the BC group with the decrease of the H_2_O_2_ concentration. This suggests that at high concentrations, H_2_O_2_ can cause oxidative stress in DEHs, while at low concentrations it can even stimulate cell growth, which is consistent with previous studies (Yue et al., 2017). Thus, the effective concentration of H_2_O_2_ to induce oxidative stress in DEHs was set in 1.5 μmol/mL. The evaluation of the anti-DHAV activity of ICA and pICA on DEHs undergoing H_2_O_2_-induced oxidative stress revealed some interesting results. DHAV infection combined with H_2_O_2_ treatment produced a stronger oxidative stress than DHAV infection or H_2_O_2_ treatment alone, indicating that DHAV can reduce cell activity by oxidative stress. In cells without H_2_O_2_ treatment, the viability of cells treated with ICA and pICA was significantly higher than that of cells from the VC group and similar to that of cells from the BC group. The H_2_O_2_ treatment led to a significant decrease in cell viability of all groups. Importantly, after treatment with H_2_O_2_, the viability of cells from the pICA-i group was not only higher than that of the VC-i group, but also higher than cells from the H_2_O_2_ and ICA-i groups (**Figure 4**). This suggests that the antioxidant activity of pICA was better than that of ICA in another aspect.

Previous experiments have shown that ICA and pICA can have a direct antiviral effect by inhibiting viral replication and release, we also investigated the effect of ICA and pICA on the TCID_50_ of DHAV after the addition of H_2_O_2_ to cause oxidative stress. The TCID_50_ of the DHAV of the VC, VC-i, ICA, ICA-i, pICA and pICA-i groups were 10^-3.626^, 10^-5.294^, 10^-2.543^, 10^-3.883^, 10^-2.289^, and 10^-3.386^, respectively. The virulence of the H_2_O_2_ treatment group was higher than that of the non- H_2_O_2_ treatment group, suggesting that the H_2_O_2-_induced oxidative stress could act synergistically with the DHAV to improve its virulence. In the drug treatment groups, treatment with ICA and pICA can reduce the virulence of DHAV regardless of whether the H_2_O_2_ was added, which is consistent with our previous assumption.

The above results indicate that ICA and pICA did have an indirect anti-DHAV effect by alleviating oxidative stress, and there are some differences between with them. To further study how the phosphorylation modification could make the antioxidant activity different from that of non-phosphorylated ICA, we studied the origin of the oxidative stress by examining, *in vitro*, the production of oxidative stress markers, such as MDA, SOD, CAT, GSH and GSH-Px (Chen et al., 2016). The same level of decrease in the MDA content was observed in the DHAV-infected DEHs treated with ICA and pICA, compared with those in the control hepatocytes infected with DHAV. We also examined the effects of ICA and pICA on the enzymatic activities of intracellular antioxidant enzymes, such as SOD, CAT and GSH-Px in DEHs. Increased SOD, CAT, GSH and GSH-Px activity inhibited by DHAV was observed in the DEHs treated with ICA and pICA. These results once again implied that the antioxidative ability of ICA and pICA might contribute to counteract the effect of DHAV. In particular, we noted that there was significant difference in the levels of SOD and GSH-Px between the ICA and pICA groups (**Table 4**). We also evaluated the relative protein expressions of SOD and GSH-Px in ICA and pICA groups by Western blot analysis. The relative protein expressions of SOD and GSH-PX in the pICA group were significantly higher than those in the pICA group (**Figure 5**), which is consistent with the results described above. Accordingly, combined with the above experimental results, we conclude that pICA exhibited better antioxidant activity than ICA by increasing the expressions of SOD and GSH-Px.

In our previous research (Xiong et al., 2015), we found that ICA and pICA can significantly reduce the amount of DHAV expression *in vivo*, and this study also found that the virulence of DHAV can be reduced by ICA and pICA *in vitro*. This proves that the ICA and pICA reagents do affect the DHAV itself. Here we propose a hypothesis that the drug affects the virus by exerting antioxidant effects. Thus, we want to prove whether the virulence of the virus is associated with antioxidant capacity. The Pearson’s correlation coefficients between the TCID_50_ and oxidative stress proved this point (**Table 5**). The TCID_50_ showed extremely high or strong correlation with the SOD, GSH-Px, CAT and MDA levels, indicating that oxidative stress is indeed related to the virus activity. Consistent with the *in vivo* results, there is a significant correlation between MDA and SOD (*P* < 0.05). These results indicate that ICA and pICA achieve anti-DHAV effects by attenuating oxidative stress.

Some differences were detected between ICA and pICA in their clinical treatment effect, and the relative indicators of oxidative stress *in vivo* and *in vitro*. In particular, the difference between SOD and GSH-Px, warrant an explanation of such difference between ICA and pICA, which could be the result of differences in the signaling pathways mediating their effects. Reactive oxygen species play key roles in the regulation of many signaling pathways, such as the mitogen-activated protein kinases (MAPKs) including p38, c-Jun N-terminal kinase (JNK) and the extracellular signal regulated kinase (ERK1/2; Usatyuk et al., 2003; Rahman et al., 2005; Jang et al., 2012). The MAPKs t are involved in the regulation of cell proliferation, differentiation and apoptosis (Garrington and Johnson, 1999; LaChapelle et al., 2007) and are activated in response to oxidative stress (Kurata, 2000; Satoh et al., 2000; Matsuzawa and Ichijo, 2005). Specifically, oxidative stress causes JNK activation, either via redox alteration of the sequestration of JNK or through inhibition of JNK phosphatase (Latchoumycandane et al., 2007). The ERK, which acts similarly to JNK in response to oxidative stress, is associated with cellular proliferation, survival, and differentiation (Conde de la Rosa et al., 2006). p38 can regulate tumorigenesis, apoptosis and autophagy by oxidative stress (Kennedy et al., 2007; Carchman et al., 2011). To identify the molecular mechanism related to the attenuation of the DHAV-induced oxidative stress by ICA and pICA treatment, we used Western blot analysis to determine the relative protein expressions of MAPKs pathway components. The result showed that the p-ERK, p-JNK, p-p38 protein expression in the DEHs of the VC group were significantly elevated compared with those of the BC group. This indicated that the MAPKs signaling pathway was indeed associated with the pathogenesis of DHAV and is consistent with the numerous previous reports on diseases that cause liver damage (Nakagawa et al., 2008; Mandrekar and Szabo, 2009; Sakai et al., 2012). In addition, as mentioned above, the treatments of ICA and pICA have shown very significant difference. For example, both can significantly reduce the phosphorylation of p38, JNK and ERK1/2, suggesting that the ICA and pICA may have antiviral effect by inhibiting the expression of the phosphorylated components of the MAPKS signaling pathway. Additionally, the p-p38/p38, p-JNK/JNK, p-ERK1/2/ERK1/2 in the cells treated with pICA were significantly lower than those in the cells treated with ICA, suggesting that the antioxidant effect of pICA is stronger than that of ICA. This indicates that, compared with ICA, pICA has better antioxidant activity associated with effective regulation of the MAPKs signaling pathway, and is consistent with *in vitro* and *in vivo* results described above.

## Conclusion

The present work indicated that oxidative stress is involved in the pathogenic mechanisms of DHAV *in vitro* and *in vivo*. ICA and pICA exhibited excellent antioxidant activity that can regulate oxidative stress related indicators and inhibit the phosphorylation of MAPKs signaling pathway components. Compared with ICA, pICA was significantly better than the former in raising the SOD and GSH-Px levels and inhibiting phosphorylation of p38, JNK and ERK1/2. Combined with the clinical data (mortality and liver damage), the results suggest that pICA still performed better than ICA to protect against the DHAV. Overall, our findings suggest that the antioxidant activity of ICA can be enhanced by its modification through phosphorylation. Finally, ICA and pICA also reduced the TCID_50_ of DHAV after oxidative stress induction by H_2_O_2_, indicating that pICA can inhibit DHAV replication more effectively than ICA. We hypothesized that pICA can alleviate the virus activity by attenuating oxidative stress thus inhibiting DHAV replication.

## Author Contributions

WX, YC, and JL conceived and designed the experiments. WX, WZ, WY, HD, KM, FY, and YC performed the experiments. WX analyzed the data. JL, DW, YH, and YW contributed to the reagents/materials/analysis tools. WX wrote the paper. All authors read and approved the final manuscript.

## Conflict of Interest Statement

The authors declare that the research was conducted in the absence of any commercial or financial relationships that could be construed as a potential conflict of interest.
